# Hypocalcemia during magnesium therapy for obstetric ICU admissions

**DOI:** 10.1186/cc12380

**Published:** 2013-03-19

**Authors:** PD Levin, A Szalat, M Vizana, CL Sprung, R Zaguri, Y Haviv

**Affiliations:** 1 Hadassah Hebrew University Medical Center, Jerusalem, Israel

## Introduction

Intravenous magnesium sulfate is commonly used in obstetric patients with pre-eclampsia. Following a case of acute symptomatic hypocalcemia we retrospectively examined a cohort of patients to investigate the frequency of hypocalcemia.

## Methods

Obstetric patients were identified from the ICU admissions database and divided into two groups - those treated with magnesium (for suspected pre-eclampsia) and those admitted for other obstetric indications (postpartum hemorrhage, infection, etc.). The baseline calcium values were compared, as well as the lowest and discharge values. Albumin and magnesium values were also compared. All comparisons used Student's *t *test.

## Results

Data were collected on 88 parturients admitted over 2 years including 40 (45%) who received magnesium and 48 (55%) who did not. Magnesium-treated women were younger (age: 31 ± 7 vs. 36 ± 5 years, *P *= 0.02). The baseline calcium concentrations were similar for the two groups (2.2 ± 0.2 vs. 2.2 ± 0.1 mmol/l, *P *= 0.85). Patients receiving magnesium had significantly higher magnesium concentrations (2.1 ± 0.4 vs. 0.7 ± 0.2 mmol/l, *P *0.001), and significantly lower calcium concentrations during therapy (1.6 ± 0.3 vs. 1.9 ± 0.3 mmol/l, *P *0.001). At discharge, the calcium levels were closer (magnesium treated 1.9 ± 0.2 vs. untreated 2.1 ± 0.1 mmol/l, *P *= 0.02). The albumin concentrations did not differ between the two groups (magnesium treated 27 ± 13 vs. nontreated 33 ± 23 g/l, *P *= 0.134). Normal values: calcium 2.15 to 2.55 mmol/l, magnesium 0.7 to 0.95 mmol/l, albumin 35 to 50 g/l.

## Conclusion

Magnesium therapy was associated with hypocalcemia. Potential causative mechanisms include a renal excretion interaction and magnesium-induced suppression of parathyroid hormone secretion. Physicians should be aware of the potential for symptomatic hypocalcemia during magnesium therapy.

